# Evaluation of the efficacy of a carbon brace (‘corset monocoque carbone respectant la respiration’ [CMCR]) preserving lung capacity to treat idiopathic scoliosis in children and adolescents: a retrospective study of 115 patients

**DOI:** 10.1186/1748-7161-7-S1-O48

**Published:** 2012-01-27

**Authors:** G Notin, JC Bernard, J Deceuninck

**Affiliations:** 1 Lecante, Lyon, France; 2 CMCR des Massues, Lyon, France

## Objectives

The purpose of this retrospective study was to investigate whether treatment with a carbon brace stops the progression of idiopathic scoliosis in children and adolescents affected by combined or thoraco-lumbar scoliosis.

## Background

The carbon brace is a single shell corset whose supports are defined according to the x rays and a 3D reconstruction [1]. Their mobility results from using carbon adjustable strips. The study was carried on a population of 115 scoliotic children whose average age is 12.5.

## Material and methods

We compared clinical features and radiolographic data at brace set-up and removal in 115 patients with combined or thoracolumbar scoliosis. The impact of the brace was evaluated in 2 subgroups according to their Risser stages. With 95 patients, a questionnaire was used to evaluate the physical and psychological tolerance of the brace.

## Results

At brace set-up, the immediate angular correction was about 50% compared to the pre-brace angle; the reduction of the vital capacity was weak. After brace removal, radiographic data showed significant improvement in thoraco-lumbar and lumbar curves of patients with combined scoliosis, although the thoracic curvature of the combined scoliosis was unchanged. No significant efficiency on the hump was observed.

## Conclusions

The CMCR can stop the progression of moderate combined or thoracolumbar scoliosis during growth, this type of orthosis provides a better outcome in terms of thoracic mobility and vital capacity, but have little efficacy on the hump. The CMCR brace is indicated for patients with flexible scoliosis. This “mobile” brace has definitely its place in the current therapeutic arsenal.
